# A Web Service-Based Framework Model for People-Centric Sensing Applications Applied to Social Networking

**DOI:** 10.3390/s120201688

**Published:** 2012-02-07

**Authors:** David Nunes, Thanh-Dien Tran, Duarte Raposo, André Pinto, André Gomes, Jorge Sá Silva

**Affiliations:** Department of Informatics Engineering, University of Coimbra, Pólo II, Pinhal de Marrocos, Coimbra 3030-290, Portugal; E-Mails: than@dei.uc.pt (T.-D.T.); draposo@student.dei.uc.pt (D.R.); afpinto@student.dei.uc.pt (A.P.); asng@dei.uc.pt (A.G.); sasilva@dei.uc.pt (J.S.S.)

**Keywords:** people centric sensing, social networks, wireless sensor networks

## Abstract

As the Internet evolved, social networks (such as Facebook) have bloomed and brought together an astonishing number of users. Mashing up mobile phones and sensors with these social environments enables the creation of people-centric sensing systems which have great potential for expanding our current social networking usage. However, such systems also have many associated technical challenges, such as privacy concerns, activity detection mechanisms or intermittent connectivity, as well as limitations due to the heterogeneity of sensor nodes and networks. Considering the openness of the Web 2.0, good technical solutions for these cases consist of frameworks that expose sensing data and functionalities as common Web-Services. This paper presents our RESTful Web Service-based model for people-centric sensing frameworks, which uses sensors and mobile phones to detect users’ activities and locations, sharing this information amongst the user’s friends within a social networking site. We also present some screenshot results of our experimental prototype.

## 1. Introduction

When putting things in perspective and considering our civilization’s history and existence so far, the Internet is nothing short of amazing. In the relatively short time it has existed it has already transformed the way our world and society work, at a very fundamental level. It has also done so at faster pace than any other massively important technological advancement. Not surprisingly, considering the social creatures we humans are, as our Internet evolved so did the means we use to communicate and interact with the people we deem close. Social networking bloomed and connected together an astonishing number of users. At the same time, sensing technologies like Wireless Sensor Networks (WSNs) are becoming more robust and viable while the sensor nodes themselves are becoming cheaper. Modern mobile phones and smartphones have also brought us mobility in our Internet access and are also a powerful computational resource, with many interesting capabilities. Mobile phones are also becoming even more powerful, common and cheaper. Never, in its entire history, did mankind have a collection of technologies that are capable of connecting and monitor such large numbers of users, simultaneously, at a global scale.

While the Web and its associated technologies will continue to evolve at increasingly faster pace, people will always live on the “real world”. Considering our social nature and the importance of “social media” throughout our history, the most likely path in our technological and social evolution is a merge between the real and the virtual: a society where people are connected not only by their physical proximity, but also by their virtual closeness. This implies the development of “people-centric” sensing systems that can acquire, process and share, in a private and secure manner, useful information about social network users. This new trend in our civilization’s evolution is still making its first steps. Our generation is on the verge of a technological revolution that will, in the next few decades, transform our society and open the path to many new market opportunities.

We are now seeing the first people-centric applications being released onto the market. In these systems, humans and their activities are the central focus of sensing and data visualization is focused towards the user’s friends and family, rather than research teams or technical staff. As the researchers of the MetroSense project suggest [1], people-centric sensing applications can be thought of as having a personal, social, or public focus. Applications with personal focus have been around for quite some time now, good examples are fitness and sports monitoring applications that use smart watches to acquire information regarding the user’s movement, heart rate and other vital signs and use this information to provide feedback on the user’s performance, like MapMyFitness [2] or Digifit [3]. Public-focused applications gather information about individual users within a metropolitan area and combine it to achieve high-level goals, such as managing the city’s resources. There are several projects on this area, namely the UCLA Urban Sensing initiative [4] and Wikicity [5]. People-centric sensing within a social context is perhaps the least explored of the three areas and the focus of our work. Applications in this area usually imply the analysis of the sensed data to detect social interactions and activities of people.

Although we already have the necessary base technology to develop these people-centric systems there are still many challenges associated with their development. Wireless Sensor Networks, for instance, are still mostly confined to research or industrial scenarios, isolated from the “real-world” and far away from useful “day to day” use. One can reason several possible causes that contributed to this situation. There are still financial and technological limitations in sensor node technology: nodes are computationally limited and still relatively expensive. Another reason is that development requires a detailed understanding of low level protocol and network, therefore requiring a great amount of effort to build new applications. This is not a particularly favorable situation in a world where the Web 2.0 and user-generated applications and content are becoming ever more important. In addition, interoperability is another difficult problem created by the many independent proprietary data formats used by different sensor networks. Although it has been proven that it is possible to implement generic communication mechanisms (both IP protocol stack (6LowPAN) and web services) on the sensor nodes, the applicability and efficiency of these technologies still needs further validation from successful real world applications. Because of their limitations, allowing directly access to sensor nodes from the Internet brings many challenges and may not be a suitable solution for all WSN applications. Therefore, it is necessary to have an infrastructure that easily, securely, and efficiently supports the integration and interoperability of sensor networks with Web 2.0 environments.

On the other hand, social networks are still very “static”. Access to social networking nowadays is becoming increasingly more mobile. It is not uncommon to see people access their smartphones to share and discuss daily experiences shortly after their occurrence, updating thoughts and responding to feedback from their friends as the situation develops and the user’s life continues. Current users can announce social events to their group of friends, share experiences through photos and comments, and show their opinions and hobbies through “likes” and their own “private wall”. Despite the general public’s interest in these social services, their current functionality does not reflect the true dynamic of people’s relationships and personal lives. Instead of being pre-determined and unique events in time, social group activities can, in fact, happen very frequently and, most of the time, spontaneously. Current systems are not capable of providing this “real-time” component to social networking, which diminishes its true potential.

With these new social trends and current technological limitations in mind, we propose SocialSense, a system that combines social networking with mobile sensing technologies to expand and provide greater functionality to social networks and other web applications. In short, this system will focus on bringing a “real-time” component to social networking, by sharing and exploring real-time information about the users’ daily actions, social activities and visited locations. By using body-area networks and mobile phones, we intend to research and develop mechanisms that focus on “people-centric sensing” and provide a seamless connection between the virtual (social networks) and the real (the user’s daily lives). This connection brings greater value not only for the user’s social experience but also for the service provider, as the new types of information available can be mined and analyzed to create more personalized and interesting product advertisement mechanisms.

A system on a chip or “system on chip” (SoC or SOC) integrates several electronic system components into a single chip. Research on the topic of people-centric sensing is highly dependent on the development of integrated and flexible, yet cheap and comfortable sensors that function with a low energy consumption. This is a challenging task since current sensor motes are still too bulky and expensive. We expect that this situation will change in the near future.

The remaining of this document is organized as follows: the next section introduces an overview of our point of view on the research topic where we present our current model and working prototype. Section 3 details our current results while the last section concludes this article.

## 2. Topic Overview and SocialSense’s Model

Bridging the real and virtual environments (Social Networks such as Facebook or Virtual Worlds such as Second Life and World of Warcraft) is a subject that has received a great deal of attention in recent scientific research, with some interesting experiences being performed in this area. The Beyond Broadcast conference, for example, not only took place in Cambridge, but also on an island in the 3-D virtual world Second Life. This was accomplished by streaming video and audio from the real-world conference to the Berkman Island in Second Life [6]. Another interesting experiment is described in [7] where a real life control panel was connected to a virtual control panel in Second Life in a way that, turning a knob on the real world one or pushing a button produced the exact same changes in the virtual replica. The work of Lifton *et al.* at the MIT media laboratory has proved to be much more complex and sophisticated. The authors coined the term “dual reality” [8,9] to indicate the ability to merge the real and virtual realities by using sensor networks. They designed several prototypes where they performed experiences in merging a real world location, the Media Lab’s third floor, and virtual worlds, in this case Second Life. One of these prototypes is described in [10] where the authors present the ShadowLab, a Second Life map of the Media Lab’s third floor animated by data collected from a network of several sensor/actuator nodes. Another dual reality implementation was the Ubiquitous Sensor Portal, a system designed for two-way cross-reality experience and communication. These portals stream information in both directions, from the user’s environment to ShadowLab, and from ShadowLab into the real world.

There are also applications of sensor nodes as means of transmitting mobility into virtual worlds. In [11] a framework is proposed which maps a sensor node to an object in Second Life. The location of the sensor mode is calculated by the framework and reflected on an avatar in Second Life which moves according to the real world movement of the node. The location of the node is calculated from the RSSI values from three or more fixed reference nodes, thus requiring a carefully designed WSN architecture.

**Figure 1.**
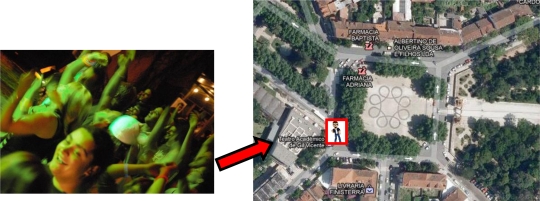
Illustration of the use of activity detection with virtual social networks. A user’s activities and location are represented in real time and become accessible amongst the user’s group of friends.

However, none of the above works could be considered “people-centric”, despite their connection to social networking. In order to better understand the applicability of people-centric sensing systems, let us consider a simple use-case. A social network user goes on a night-out with some of his friends to a local nightclub. He has an activity detection system that runs on his smartphone, possibility accompanied by several small body-area wireless sensors (in the form of a small bracelet, for example). The small sensors in his body directly communicate with the smartphone through a body-area wireless sensor network and share information regarding several conditions, such as his movement (accelerometer) and heartbeat. The sensors can also detect environment factors such as the temperature. The smartphone runs activity detection algorithms that combine the sensory information (for example, an increase in heart rate, temperature and movement) to discern what the user currently doing, in this case, dancing. The smartphone accesses the Internet and communicates with the user’s preferred social network to represent information about the user’s current activities to his trusted group of friends. The information can be represented through a virtual representation of the user and the environment. If the user’s friends also use the same system, it is possible to correlate their close physical proximity with activity detection and deduce that the group is currently engaging in a social group activity (they are all dancing in a nightclub). People who belong to this same group of friends who are still at home and were indecisive as to whether or not go to the nightclub can now have an idea of who is attending the social event and where it is happening, and use this information to make their decision. Figure 1 illustrates the use-case above.

The above use-case is a good example of how a people-centric sensing system could be a part of a regular person’s social life. The term “people-centric sensing” is used by the MetroSense project [12] to describe a vision where the majority of network traffic and applications are related to sensor and actuator data, applied to the general populace. The MetroSense project envisions collaborative data gathering of sensed data by individuals, facilitated by sensing systems comprised of cheap and easily accessible mobile phones and their interaction with software applications. As a part of the MetroSense project, the authors of the work in [13] proposed the use of sensors embedded in commercial mobile phones to extrapolate the user’s real-world activities that in turn can be reproduced in virtual settings. The authors' goal was to go further than simply representing locations or objects and provide virtual representations of humans, their surroundings and their social interactions. The system prototype implementation proposed was named CenceMe and allows members of social networks, namely Second Life, to process the information sensed by their mobile phones and use this information to extrapolate information about the user’s surroundings and actions.

The state of the art in the area still represents only the first steps for these people-centric sensing systems. There is plenty of room for improvement in various areas, encouraging future research. In fact, creating such systems still presents several technical challenges that we intend to address with our research, namely:

- **Privacy** Exchanging sensitive information such as user’s vital signs, location and current activity is a task that has strict privacy requirements. Privacy is a requirement that affects all application layers: from the sensor’s wireless communication to the exchange of middleware messages. During our research we intend to employ effective privacy mechanisms that ensure the safety of sensitive information.
- **Managing intermittent connectivity** Reliability of wireless sensor networks is very important in order to create systems that are robust and stable. However, due to the wireless nature of mobile phone and WSNs communication, there is always a risk of intermittent connectivity due to environment interference, power shortages or communication range. Robust people-centric sensing systems should be capable of maintaining an illusion of smooth and continuous people tracking. Offline mechanisms that can estimate a user’s current activity and location even when there is a shortage of fresh data can maintain an illusion of continuous monitoring in adverse conditions. These offline mechanisms can also be employed in data mining and analysis, since their function is to find patterns in the data.
- **Detection of activity** Based on previous work in the area, our research will attempt to improve and design resource aware algorithms that use both the mobile phone and wireless sensors and are directed specifically towards the representation of activities in social networking sites and virtual communities. At the same time, we will also seek interesting and engaging ways of representing user activities and environmental data, through the use of virtual representations of users (virtual avatars).

Aside from these technical challenges from an application point-of-view, research on the topic of people-centric sensing is also highly dependent on cheap and comfortable sensors that have a decent battery life. One of the current obstacles in our research is a lack of extremely lightweight and small sensors that can be used in a ubiquitous way, that is, the user forgets that he is using them. The area of SoC is extremely important for the development of new sensor devices that are both extremely flexible, comfortable and with a good battery-life. For example, in [14] the authors have published the design methodology and a demonstration of a silicon chip that has all the necessary characteristics for medical diagnostics, environmental monitoring and personal connectivity systems. More recently, the authors in [15] describe the design, implementation, and verification of a system-on-chip aimed to play the role of a general purpose processor for a wireless body area sensor network node. This a very interesting work, since body area sensor networks are the basis of people-centric sensing. Continuous research on the field brings further innovations; the work in [16] shows miniaturized “plug & play” sensor nodes that can collect vital physiological information and wirelessly transmit it to the system database.

We intend to design a model based on innovative solutions that effectively address all these technical challenges. Small and discreet wireless sensors allow for more diverse kinds of vital-signs data to be used, which permit the creation of accurate activity detection systems. Activity detection amongst groups of friends can be correlated to automatically create social events that can be shared in Social Networks. Localization techniques can be used to permit users to locate their friends in big social events. Offline inference techniques can provide an illusion of seamless connection between the real and the virtual, by trying to infer current location and activity even when there is no fresh data due to a connectivity problem. All of these research approaches attempt to expand the features of social networking sites and increase their functionality beyond the simple sharing of photos and comments.

**Figure 2.**
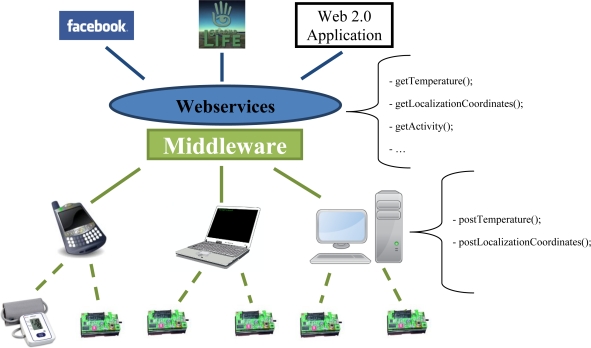
SocialSense’s current architecture and some examples of used services.

The research herein proposed is based on a testbed already implemented in our labs. In our current model, Wireless Sensor Networks (WSNs) are data and functionality providers, managed by an integration middleware. This platform is called SocialSense and provides a set of services for consumption by external environments using web service APIs. This model facilitates the integration and interoperability of WSNs with virtual world environments, social networks and other web 2.0 applications. Our approach is based on a hybrid gateway-based model that supports both proactive and reactive communication. In proactive communication the sensor nodes periodically collect data and send it to the middleware for processing. In reactive communication, the end users send data/functionality requests to the middleware which then uses the sensor node’s data to react accordingly. A simplified illustration of our current prototype’s architecture is depicted in Figure 2. The major difference between our basic architectural solution and CenceMe [13] is our reliance on wireless sensors external to the mobile phone, a central middleware and the exposure of our system’s data and functionality through web services. Wireless sensors are becoming increasingly smaller and comfortable to use, so their addition is an advantage over CenceMe, since we have more different types of data that can be used to more accurately detect human activities. A centralized middleware allows us to have more control over client requests and data, facilitating the future implementation of robust privacy mechanisms. RESTful web services open our system’s functionality to the public, promoting the re-use of wireless sensor deployments and the creation of new web applications by third-parties outside the scope of our project.

Our model connects the middleware with the sensor networks through several entities called “proxies”. They receive data packets from the sensor nodes and perform earlier processing tasks, forwarding the information to the middleware for more advanced management processing. In addition, they may also handle specific commands and requests coming from the middleware to satisfy client applications, by performing queries to the sensor nodes. Proxies also include a few components that support mobility. One of the main advantages of this modular approach is that it can easily offer mobility, localization and scalability. Proxies are designed to be lightweight, so that they may be installed on both computers/laptops and modest or limited devices such as smartphones. Each proxy can handle communication with several wireless sensor networks and all proxies eventually connect to the central middleware service.

The middleware gateway exposes the functionality and data of sensor networks as web services. It is the central part of our framework, the basis for the integration and interoperability between sensor networks and other Web 2.0 environments. This approach facilitates the development of applications using sensor networks (users simply use a web service, instead of programming the wireless network), increases the diversity of possible web applications and promotes the reuse of WSN deployments and software code. In addition, data coming from the sensor networks can easily be stored in a cloud data warehouse for further processing. The middleware contains the necessary intelligence to process messages coming from different sensors and requests coming from different users and applications. It also contains the necessary interfaces to proactively send data to different Web 2.0 applications.

The activity detection module is responsible for processing sensor data and running algorithms that are capable of classifying current user activity. Current prototype versions of this module use accelerometer data to perform the detection of very simple activities, such as walking or running. These earlier versions were also implemented as part of the middleware service, but in the future the module will be moved onto a mobile proxy (possibility a smartphone) in order to promote mobility and scalability (signal processing tasks are to be performed by the user’s own hardware). As our research advances we will explore the use of more types of data and attempt to design more efficient and accurate mechanisms for detecting activities.

Privacy is an area still under very active research and implementation. We are currently implementing a privacy mechanism based on TARF, a trust-aware routing framework for WSNs presented in [17]. TARF is an energy-efficient, highly scalable, and well adaptable solution that integrates both trustworthiness and energy efficiency in making routing decisions. TARF’s privacy mechanism is based on a “Trust level” measurement that represents a node’s opinion of his neighbor’s level of trustworthiness. Neighbors with trust levels below a certain threshold are excluded as candidates for data routing operations. When an intruder forges the identity of a legitimate node, at first, the intruder will be able to deceive its neighbors, but as the legitimate node broadcasts information about undelivered packets, the intruder’s neighbors will start to downgrade its trust level and the intruder is eventually left out of the network. Our work intends on improving on TARF in a sense that TARF only allows for the downgrade of a neighbors’ trust level, while we intend to create a more dynamic system that can both increase or decrease a node’s trustworthiness depending on the situation. When thinking in terms of Social Network access there are also several important issues regarding privacy. Regarding Facebook, in particular, there are several efforts that try to expose its most relevant privacy and security flaws. For example, “The Very Unofficial Facebook Privacy Manual” [18] shows several privacy issues present Facebook, such as the fact that when you send a message to someone on Facebook they get limited access to your profile or when someone adds you as a friend and you haven’t said yes or no to them, they can see your updates in their home feed. These types of issues can be avoided and circumvented for SocialSense data, since our solution is independent from any particular social network.

Intermittent connectivity and interference avoidance are areas that were already a subject of previous work within our research group. Namely, the work presented in [19] addresses interference issues on channels of IEEE 802.15.4 wireless networks. The study shows that it is difficult to predict the quality and stability of different wireless channels in sensor networks as the environment conditions greatly influence network performance and provides an experimental evaluation of the best channels for IEEE 802.15.4 networks. While the results and experience gained from our previous evaluations were already applied in our current prototype, research in intermittent connectivity will continue to be a part of future deployments.

SocialSense was also thought to be able to support the use of several medical devices. In our current implementation, we use a small heart beat monitor (also depicted in Figure 2), whose communication is based on the Bluetooth protocol. The use of these “external” devices requires an intermediate device (mobile phone or wireless node) that supports both the external protocol (Bluetooth) and communication with the middleware.

From the beginning, our vision was not to just propose a model for the integration of WSNs with web environments, but to expand this model towards people-centric sensing and to include mechanisms to provide location, vital signs and activity detection, using mobile phones and wireless sensor nodes as base hardware. We intend to offer a robust platform that offers environmental and personal information and allows this information to be combined in useful ways for both social networking users and service providers.

## 3. Results and Discussion

Our most recent implementation of the model supports 6LowPAN as well as traditional WSNs. Communication between proxies and middleware is based on TCP/IP and RESTful web services, currently supporting XML and JSON as the data formats. REpresentational State Transfer (REST) [20] is a web service design idiom that uses a stateless client-server architecture in which the web services can be identified by URLs. Web service clients wanting to use these services have to resort to a small set of remote methods that are very similar to the ones present in HTTP, such as GET and POST.

**Figure 3.**
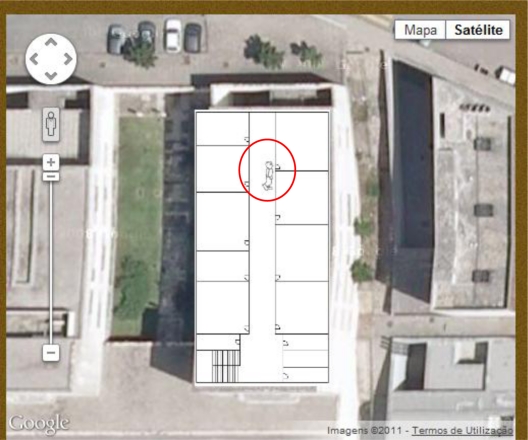
Current Web Interface displaying a user’s location and a simple avatar (circled red).

The communication between proxies and sensor nodes is currently implemented independently using any protocols that are appropriate, in order to allow interoperability between different networks. Web services at the “bottom level” are used by the proxies for posting new data to the middleware. The web services provide basic functionality for storing data (which can be sensory or localization data) within the system in a private database. Examples of such “low level” web services called by proxy devices are the “postTemperature();” and “postLocalizationCoordinates();” services, although there are others of similar functionality. “Higher level” web services are used by web interfaces and applications to retrieve ready-to-use, processed data from the middleware. Our own web interface (that serves as a link between our framework and Facebook) uses several web services, such as “getTemperature();”, “getLocalizationCoordinates();” and “getActivity();” in order to update the user’s avatar position within the map of our testbed, as shown in Figure 3.

These web services are responsible for retrieving the most recent data from our database regarding a user’s current location, vital signs or activity. In addition to the web services provided by our own middleware, our current web interface also integrates with the Google Maps API in order to display the users’ location and activity by overlaying a map of our testbed and a small avatar.

The interface allows users to select those who will have access to their own activity and location, and we implemented a login-based security mechanism to access data in our middleware. This login-based mechanism relies on Facebook’s own “access token” mechanism: when a user accesses our application, a web page asks for permissions to fetch various types of data (the user’s friends, Facebook ID, *etc.*). In case the user accepts these terms, an “access token” is generated by Facebook and sent to our middleware by the user’s browser through HTTPS. This access token is then used by our middleware to communicate with Facebook and fetch the user’s Facebook ID, which is then compared to the existing IDs in our database. In case the ID exists on the database, the user is given access to sensory data, otherwise, our webservice returns nothing. Thus, only users registered within our system can fetch relevant data. The middleware supports a localization services based on the RSSIs of the received wireless packets and a trilateration algorithm.

We developed a simple activity detection based on the data collected from the accelerometer sensor of Zolertia Z1 motes [21]. With the accelerometer’s movement speed data, we perform simple signal frequency analysis tasks to classify the user’s activity as “moving” or “not moving”. Our approach is similar to the one described in [22], frequencies above the ones resulting from human walking motion are filtered out and a simple sum of the Fast Fourier Transformation coefficients is performed in order to get a rough idea of the intensity of the movements being experienced. This intensity is used to determine if the user is either walking or stopped. At the same time, localization can be used to further distinguish the type of movement; for example, if the user’s movement’s speed is less than 6 km/h then the system determines that the user is “walking”. Otherwise, the user is running on foot or driving car, depending on the scenario. This classification scheme is extremely simple and results from our early efforts in establishing a working prototype. Future work will result in more advanced classification schemes.

**Figure 4.**
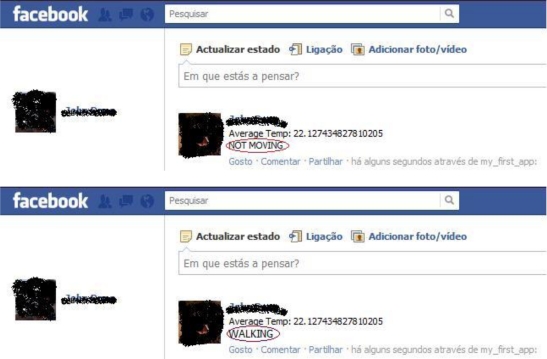
Activity and sensory information are displayed on the Facebook user’s personal wall.

In order to integrate activity detection with social networking, we developed a mashup application that combines the services provided by our platform (through the middleware web services) with the Facebook platform. The mashup periodically collects user activity and sensor data and publishes them into the user’s personal wall. The results of this integration are shown in Figure 4.

The sensors used for our application were general-purpose Zoleria Z1 motes. These sensors are unfortunately bulky and not wearable. Future iterations of our work would benefit from the use of more comfortable, smaller and application-specific sensors stemming from current research in the field of SoC. A good example of an interesting sensor for people centric applications is the UP bracelet [23] from Jawbone, which provides motion sensing and heat beat monitoring. While the concept is very good, the bracelet still presents some limitations. We believe that people-centric sensing systems should focus on using common technology (mobile phones) and cheap and flexible technology, in order to promote the creation of interesting applications in a Web 2.0 context. The UP bracelet is quite expensive (aprox. 100 euros) and the diversity of measurements is low (only measures movement and heartbeat, which is quite common and does not justify the high pricing). It is also part of an integrated and closed monitoring system, thus limiting it’s applicability in new people-centric systems. These limitations make it less interesting for both research and the general populace.

**Figure 5.**
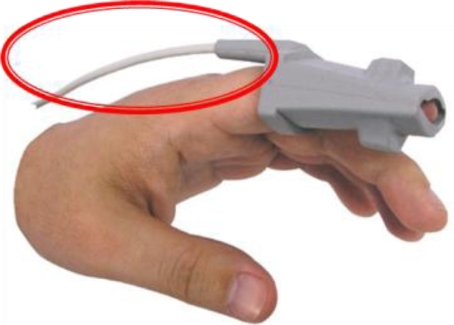
SpO_2_ sensor. The sensor’s cable and size make it very uncomfortable to use in sports or social interactions [24].

From our point of view, ideal sensors for people-centric sensing applications must be very comfortable and lightweight and provide as many measurements as possible. This is a problem, since many interesting vital signs can only be measured with cumbersome technology. To better explain our vision, let us consider a people centric sensing system that has “multiple” solutions. The “basic” solution has only the most important hardware (a bracelet with heart beat and accelerometer, for example) that acts as a direct bridge to the user’s mobile phone. However, users can acquire more sensors that provide greater capability. A “sports” solution, for example, can provide more sensors for a better assessment of physical exercise. Oxygen saturation (SpO_2_) is an interesting measurement for this case, since it represents the amount of oxygen that dissolved or carried in the user’s bloodstream. However, if we wanted to use this measurement, we would have to depend on cumbersome sensors that are too big to be comfortable and usable in a social context, as the one shown in Figure 5.

**Figure 6.**
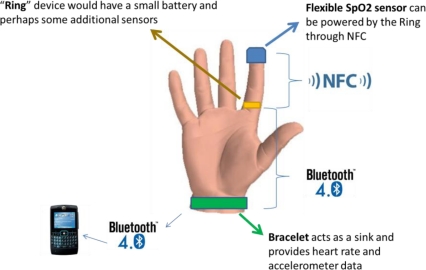
Possible combination of hand-worn sensors that can be used in a people-centric sensing application.

From our understanding, there are several reasons that contribute to the SpO2 sensor’s large size and cabling, namely their need for batteries. One solution to this problem could be the use of Near Field Communication (NFC) technologies in order to remove the need for both cabling and batteries, drastically reducing the sensor’s overall size. Recently, STMicroelectronics has introduced a 16-kbit memory NFC chip designed to employ energy harvesting technology to power up small devices, thus eliminating the need for an internal power supply [25]. At the same time, NFC can be applied for wireless communication, thus removing the need for cabling. Thus, an interesting SoC challenge would be to combine NFC with a SpO2 sensor, providing a more comfortable solution for people-centric systems. In Figure 6 we present an interesting combination of sensor devices and communication technologies that could provide an interesting solution for activity detection that is relatively comfortable, small and, perhaps, even “fashionable”:

Since NFC has a very short range (max. 20 cm, but typically only a very few cm), a “Ring” device could act as a wireless bridge between the SpO2 sensor and the bracelet. This would require embedding Bluetooth Low Energy and NFC onto a single device, while maintaining the overall price as affordable as possible. This solution would be extremely interesting from our point-of-view, since it would provide many different measurements while remaining relatively comfortable for the average user.

## 4. Conclusions

Although the obtained results presented in this work are very simple, they prove that it is feasible to seamlessly merging physical and virtual world and develop new applications by accessing openly exposed sensory information through webservices. They also show that it is feasible to detect users’ activity based on the data from sensors. This work represents our first steps into our research on people-centric sensing.

The sensors used on our application prototype were general purpose wireless sensors. These are not adequate for real-world deployments of people-centric sensing systems. Such systems benefit from small sensors that are both comfortable to wear and energy efficient. Future developments in the field of SoC will prove to be important for the development of sensors that are specifically oriented for these kinds of systems. We expect that future sensor technology will become cheaper and smaller, making people-centric systems more attractive to the general populace.

## References

[1] Campbell A.T., Eisenman S.B., Lane N.D., Miluzzo E., Peterson R.A., Lu H., Zheng X., Musolesi M., Fodor K., Ahn G.S. (2008). The rise of people-centric sensing. IEEE Internet Comput.

[2] Mapmy: Fitness. http://www.mapmyfitness.com/imapmy/wahoo/.

[3] Zahradnik F. Digifit: Wireless Heart Rate Monitor Plus Fitness, Training Apps for iPhone. http://gps.about.com/od/mobilephonegps/a/digifit-iPhone-heart-rate-review.htm.

[4] Urban Sensing. http://urban.cens.ucla.edu/.

[5] Calabrese F., Ratti C., Kloeckl K. (2009). Wikicity: Real-time location-sensitive tools for the city. Handbook of Research on Urban Informatics: The Practice and Promise of the Real-Time City.

[6] (2008). The Conference Goes Virtual: Second Life. http://www.beyondbroadcast.net/blog/?p=37.

[7] (2004). Real Life Control Panel for Second Life. http://channel3b.wordpress.com/2007/01/24/real-life-control-panel-for-second-life/.

[8] Lifton J. (2007). Dual Reality: An Emerging Medium.

[9] Lifton J., Paradiso J.A. Dual reality: Merging the real and virtual.

[10] Lifton J., Laibowitz M., Harry D., Gong N.-W., Mittal M., Paradiso J.A. (2009). Metaphor and manifestation—Cross-reality with ubiquitous sensor/actuator networks. IEEE Pervas. Comput.

[11] Tran T.D., Silva J. A Framework for Integrating WSNs and External Environments.

[12] Metrosense Project. http://metrosense.cs.dartmouth.edu/.

[13] Musolesi M., Miluzzo E., Lane N.D., Eisenman S.B., Choudhury T., Campbell A.T. The second life of a sensor—Integrating real-world experience in virtual worlds using mobile phones.

[14] Wang L., Aydin N., Astaras A., Ahmadian M., Hammond P.A., Tang T.B., Johannessen E., Arslan T., Beaumont S.P., Flynn B.W. A sensor system on chip for wireless microsystems.

[15] Stamenkovic Z., Panic G., Schoof G. A system-on-chip for wireless body area sensor network node.

[16] Saeed A., Faezipour M., Nourani M., Tamil L. Plug-and-play sensor node for body area networks.

[17] Zhan G., Shi W., Deng J. (2010). Tarf: A trust-aware routing framework for wireless sensor networks. Lect. Notes Comput. Sci..

[18] Alcorn A. (2010). The Very Unofficial Facebook Privacy Manual. http://netsavoir.com/2010/10/27/facebook-privacy-manual/.

[19] Tran T.-D., Silva R., Nunes D., Silva J. (2010). Characteristics of channels of IEEE 802.15.4 compliant sensor networks. Wirel. Pers. Commun.

[20] Tyagi S. (2006). Restful Web Services. http://www.oracle.com/technetwork/articles/javase/index-137171.html.

[21] Zolertia Z1 platform. http://www.zolertia.com/ti.

[22] Welbourne E., Lester J., Lamarca A., Borriello G. (2005). Mobile context inference using low-cost sensors.

[23] Jawbone webpage. http://jawbone.com/up.

[24] Reusable SpO2 Sensors. http://www.sensoronics.com/images/reusable-spo2-sensors-large.jpg.

[25] Clark S. (2011). NFC goes green: New ST chips use energy harvesting to replace the need for batteries. http://www.nfcworld.com/2011/11/08/311126/nfc-goes-green-new-st-chips-use-energy-harvesting-to-replace-the-need-for-batteries/.

